# Expression of New Gene Markers Regulating Protein Metabolism in Porcine Ovarian Granulosa Cells In Vitro

**DOI:** 10.3390/ijms262411942

**Published:** 2025-12-11

**Authors:** Krzysztof Data, Wiesława Kranc, Małgorzata Blatkiewicz, Dominika Domagała, Julia Niebora, Piotr P. Chmielewski, Artur Bryja, Izabela Berdowska, Agnieszka Żok, Magdalena Kulus, Jakub Kulus, Teresa Wysocka, Robert Z. Spaczyński, Hanna Piotrowska-Kempisty, Paul Mozdziak, Bartosz Kempisty, Paweł Antosik, Dorota Bukowska, Mariusz T. Skowroński

**Affiliations:** 1 Division of Anatomy, Department of Human Morphology and Embryology, Faculty of Medicine, Wroclaw Medical University, 50-368 Wroclaw, Poland; 2 Department of Anatomy, Poznan University of Medical Sciences, 60-781 Poznan, Poland; 3 Department of Histology and Embryology, Poznan University of Medical Sciences, 60-812 Poznan, Poland; 4 Department of Medical Biochemistry, Faculty of Medicine, Wroclaw Medical University, 50-368 Wroclaw, Poland; 5 Division of Philosophy of Medicine and Bioethics, Department of Social Sciences and the Humanities, Poznan University of Medical Sciences, 60-812 Poznan, Poland; 6 Department of Veterinary Surgery, Institute of Veterinary Medicine, Nicolaus Copernicus University in Torun, 87-100 Torun, Poland; 7 Department of Diagnostics and Clinical Sciences, Institute of Veterinary Medicine, Nicolaus Copernicus University in Torun, 87-100 Torun, Poland; 8 Department of Anatomy and Histology, Collegium Medicum, Institute of Health Sciences, 65-046 Zielona Gora, Poland; 9 Center for Gynecology, Obstetrics and Infertility Treatment Pastelova, Pastelowa 8, 60-198 Poznan, Poland; 10 Collegium Medicum, University of Zielona Gora, 65-046 Zielona Gora, Poland; 11 Department of Toxicology, Poznan University of Medical Sciences, 60-812 Poznan, Poland; 12 Department of Basic and Preclinical Sciences, Institute of Veterinary Medicine, Nicolaus Copernicus University in Torun, 87-100 Torun, Poland; 13 Prestage Department of Poultry Science, North Carolina State University, Raleigh, NC 27695, USA; 14 Physiology Graduate Faculty, North Carolina State University, Raleigh, NC 27695, USA; 15 Department of Obstetrics and Gynecology, University Hospital and Masaryk University, 602 00 Brno, Czech Republic

**Keywords:** metabolism, signaling pathway, cell signaling, gene expression, follicular granulosa cells, in vitro culturing, oocyte

## Abstract

During oocyte maturation, granulosa cells (GCs) respond to fluctuating hormone levels in the ovary. The study aims to reveal metabolism and activity patterns of isolated and cultured GCs, reflecting in vivo processes. A downregulation of GARNL3 and ARRDC4 across all time points (48 h, 96 h, and 144 h) suggests reduced cell signaling and response to external stimuli, which may be related to the isolation and in vitro culturing of GCs from the complex ovarian microenvironment. The consistent elevation of LOX underscores its role in extracellular matrix (ECM) cross-linking, crucial for oocyte quality, whereas FN1 and ITGB3 highlight cellular adhesion and ECM interaction during adaptation to in vitro conditions. The study further demonstrates that ANKRD1 and SLC1A1 are upregulated over time in vitro, indicating cellular differentiation and metabolic alterations. Furthermore, proteoglycan and MAPK signaling pathways are identified as key players in cell-to-cell and cell-to-ECM interactions. GSEA revealed heightened activity in vasculature development, the TGF-β signaling pathway, cell development, and lipid response. The findings suggest that while GCs in vitro mimic in vivo processes related to ECM remodeling and oocyte development, they also exhibit a tendency towards aging. The research emphasizes that isolated GCs in vitro exhibit time-dependent activity shifts related to cellular differentiation, ECM remodeling, and lipid metabolism, which also have implications for the understanding of reproductive physiology and pathologies.

## 1. Introduction

Granulosa cells (GCs) are essential components of ovarian follicles, which play a crucial role in reproductive biology. These somatic cells surround the developing oocyte, providing proper environmental conditions for oocyte maturation [1,2]. The role of the GC is to structurally support, and mediate transport of the signaling molecules, proteins and hormones. As the supporting cells, GCs are responsible for providing most of the nutrients required for oocyte development [3,4]. During folliculogenesis, GCs undergo specific significant morphological and functional changes triggered via paracrine and endocrine factors. A prominent GC’s response is regulation by follicle-stimulating hormone (FSH), which triggers FSH receptor, which controls proliferation, differentiation, but especially protein secretion [5,6,7]. The wide network of synthetized and secreted proteins fulfills many metabolic actions, especially modulating FSH secretion through the production of estrogen [8]. The mediating relationship between GCs and oocyte occurs continuously, using protein signaling pathways as growth differentiation factor 9 (GDF9), and bone morphogenetic protein 15 (BMP15), mitogen-activated protein kinases (MAPK), Integrin beta-1 (ITGB1), and many others [9,10,11]. Granulosa cells undergo progressive changes in response to pituitary-derived hormones during the menstrual or estrous cycle. During luteal phase, post-ovulation, they transform into lutein cells involved in progesterone synthesis, which is necessary for maintaining pregnancy [12]. A better understanding of metabolic process, expression profiles, and responses to various signaling pathways provide advancing development of reproductive biology and GCs as a potential therapeutic factor for infertility treatments [13,14,15]. A key metabolic process for GCs is the metabolism of proteins, including proteoglycans, family of heavily glycosylated membrane proteins anchoring cells in extracellular matrix (ECM), and supporting intercellular connections [16,17]. Proteoglycans participate, informing the cumulus–oocyte complexed extracellular matrix. Therefore, proper proteoglycan synthesis and secretion is crucial for further oocyte development [18]. Proteoglycans influence follicle dynamics by modulating interactions between granulosa cells, theca cells, and the oocyte [19,20]. Proteoglycans such as decorin and perlecan bind growth factors such as FGF and TGF-beta, regulating their activity and promoting processes essential for oocyte maturation, including cellular proliferation, differentiation, and adhesion [21,22,23]. The proteoglycan metabolism predisposes the development of neoplasms in the ovary [24]. Proteoglycans are composed of a core protein with attached one or more glycosaminoglycan (GAGs) chains. GAGs are divided into two types: sulfated and nonsulfated. Sulfated GAGs, such as dermatan sulfate, heparan sulfate, keratan sulfate, and heparin, are mostly found in living organisms. Nonsulfated GAG, hyaluronic acid, is not bound to a core protein, but it is a key part of the ECM [25]. Proteoglycans are crucial regulators of tissue mechanical properties, ECM modulation, cell signaling, and protein metabolism in normal-health tissue [26].

Processes of GCs’ metabolism involve various pathways that supply energy, regulate hormone synthesis, and support follicle growth. Plenty of metabolism processes are well-known and described, including metabolism of glucose, amino acids, and steroidogenesis [27,28,29,30,31,32,33]. Individual metabolic processes constitute a highly hierarchical group, the components of which often belong to multiple categories, cross-linking and positively or negatively regulating subordinate and parallel processes. Thus, phosphonoacetate metabolic processes or phosphate-containing compound metabolic processes are equally assigned to phosphorus metabolic processes [34]. The basics of phosphorus metabolic processes are chemical reactions and pathways that are involving phosphorus or compounds containing phosphorus, which are crucial i.a. during protein metabolism [35]. Protein metabolism and also protein–protein interaction networks and pathways are key regulators in whole cell metabolism, regulating holistic cell fate, especially during differentiation and development of maturing oocyte and surrounding cells. An example of a versatile signaling pathway is mitogen-activated protein kinase (MAPK), engaging various proteins, depending on the triggering factors. MAPK regulates cellular proliferation, differentiation, development, and apoptosis. A well-organized regulation of the pathway is crucial for proper functioning of the developing tissues [36,37].

In vitro culture provides a tool for studying metabolism and its changes in granulosa cells, and during culture cell metabolism changes over time, influenced by cell–cell interactions, cell–environment interactions, and also parameters of used cell culture reagents. In vitro culturing significantly influences the regulation of protein metabolism and activity in cells by altering their metabolic environment and the availability of key factors [38,39,40]. In vitro conditions, such as the type of serum, can impact the fatty acid and protein metabolism of cells. The aim of the study was to measure and classify marker genes of protein metabolism, as well as those related to intra- and extracellular activity of GCs.

## 2. Results

**Figure 1:**
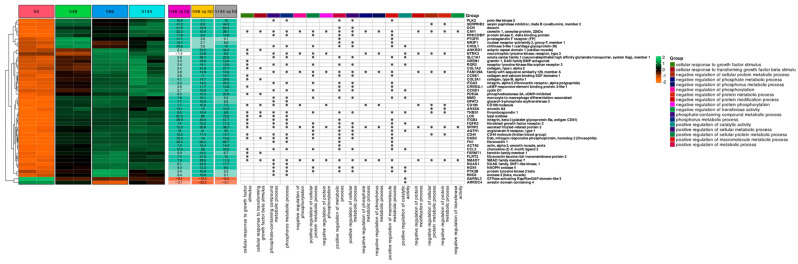
Heatmap featuring hierarchic clustering of differentially expressed genes in 48, 96, and 144 h of experiment compared to control (0 h). Dark dots represent genes from the most significantly enriched ontological groups based on the lowest adjusted *p*-values. Expression values are scaled by rows and presented as colors and range from orange (low expression) to green (high expression).

A comprehensive analysis of microarray data to identify and interpret differentially expressed genes (DEGs) using the “Limma” algorithm for identifying DEGs. The heatmap for hierarchical clustering visualizes patterns of gene expression across different conditions (Figure 1). Gene expression profiling revealed activation patterns. Only two genes, GARNL3 and ARRDC4, disclose suppressed expression during the experiment. At the first time point of the experiment (48 h), the highest fold change relative to the control freshly isolated cells was observed for LOX, SERPINB2, FN1, ITGB3, and CHI3L1 (fold change 67.3; 41.2; 35.4; 29.9; and 29.6, respectively). The second time point (96 h) revealed enhanced expression of ANKRD1, LOX, FN1, SLC1A1, and COL1A2 (fold change 91.2; 89; 66.2; 36.7; and 31.8, respectively). The last of the analyzed time points (144 h) indicated that the five mostly elevated genes were LOX, FN1, DCN, ITGB3, and SERPINB2 (fold change 75.6; 68.7; 63.4; 57.7; and 44.9, respectively). The highest number of differentially expressed genes were related to phosphorus metabolism. Meanwhile, the lowest number of differentially expressed genes were involved in the negative regulation of transferase activity and the negative regulation of the protein modification process.

**Figure 2:**
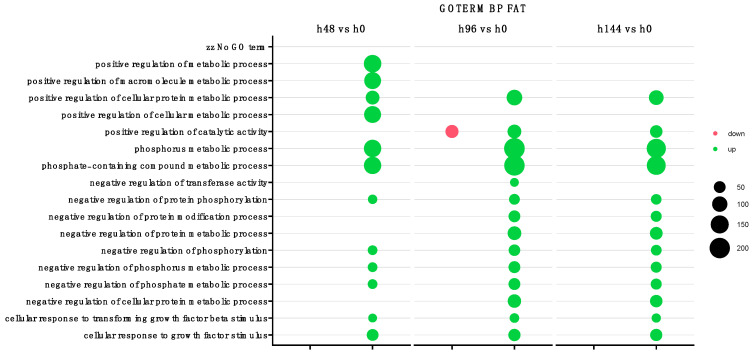
The overrepresented gene sets in DAVID GO PB DIRECT annotations database illustrated as a bubble plot, derived from comparisons in gene expression profiles between 48 h, 98 h, and 144 h vs. control (0 h). Only the GO groups above the establisher cut-off criteria (*p* with correction < 0.05, a minimal number of genes per group > 2) are depicted as color bubble. Each bubble’s size corresponds to the number of differentially expressed genes associated with the GO biological process terms. The transparency of the bubble represents its *p*-value, with greater transparency indicating proximity to the *p* = 0.05 cut-off value. Green bubble represents overexpressed genes, while red bubbles signify downregulated genes.

**Figure 3:**
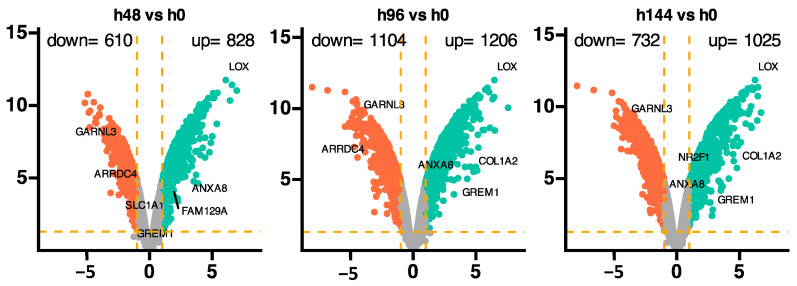
Volcano plots depicting general expression profiles, with each dot representing the mean expression of an individual gene obtained from a normalized microarray study. Orange dotted lines, serving as cut-off values, were determined by the criteria |fold change| = 2 and *p*-value = 0.05. Genes surpassing these cut-off lines are identified as differentially expressed and are represented as orange dots (downregulated) and green dots (upregulated). The total counts of up- and downregulated genes are provided in the top right and top left corners, respectively. Additionally, the symbols for the most differentially expressed genes from each comparison are highlighted on the plots.

Furthermore, the gene ontology (GO) biological processes analysis revealed 8 of 17 processes to be significantly impacted during all time points that were evaluated (Figure 2). The highest number of enriched genes were noticed for phosphorus metabolic, phosphate-containing compound metabolic, and positive regulation of cellular protein metabolic processes. The analysis revealed only one suppressed process for 96 h of experiment, compared to control, which was positive regulation of catalytic activity. The volcano plots for the overall analysis, representing the relationship between statistical significance (*p*-values) and fold changes for each gene (Figure 3). For the analysis of 48 h of the experiment, 610 downregulated and 828 upregulated genes compared to the control group were revealed. The most inhibited genes were *GARLN3* and *ARRDC4*, while the most activated were *ANXA8*, *SLC1A1*, *FAM129A*, and *GREM1*. The comparison of 96 h of experiment to control showed 1104 inhibited (*GARNL3*, and *ARRDC4*), and 1206 activated genes (*LOX*, *COL1A2*, *ANXA8*, and *GREM1*). Analysis of the last time point (144 h) indicates 732 inhibited (*GARNL3*) and 1025 enhanced genes (*LOX*, *COL1A2*, *NR2F1*, *ANXA8*, and *GREM1*). To summarize, in all analyzed time points, the expression of *LOX*, *ANXA8*, and *GREM1* was enhanced, while the expression of *GARNL3* and *ARRDC4* was suppressed.

**Figure 4:**
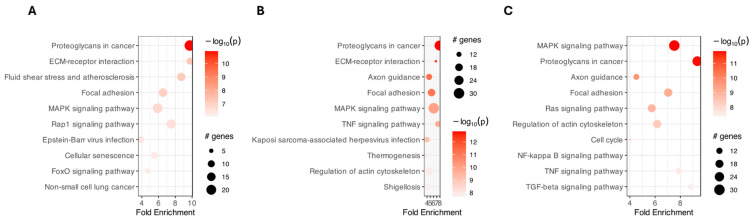
The bubble chart illustrates the enrichment results from 48 (**A**), 96 (**B**), and 144 (**C**) hours of experiment compared to control (0 h). The horizontal axis illustrates fold enrichment values, and the vertical axis represents enriched terms. Each bubble’s size represents the number of significant genes linked to the specific enriched term. The color of the bubbles is determined by the −log10 (lowest *p*-value), with shades closer to red indicating a higher level of significance in the enrichment. (symbols; # genes (number of genes)).

**Figure 5:**
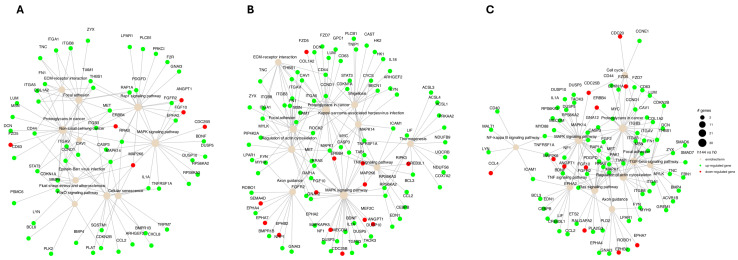
The graph illustrating term–gene relationships for significant genes associated with enriched terms at 48 (**A**), 96 (**B**), and 144 (**C**) hours of the experiment compared to the control. Node sizes are proportionally plotted based on the number of genes within a term, with node color indicating upregulated genes (green) and downregulated genes (red).

PathfindR was used to identify enriched pathways and active subnetworks in a protein–protein interaction network (Figure 4 and Figure 5). We indicate that in all analyzed groups, the term “proteoglycans in cancer” was enriched (Figure 4). Furthermore, the “MAPK signaling pathway” had the highest number of involved genes across all analyzed groups. Additionally, the connections between terms and significant genes were explored to gain deeper insights into the complex relationships and regulatory networks within the biological processes identified in the preceding analysis (Figure 5).

**Figure 6:**
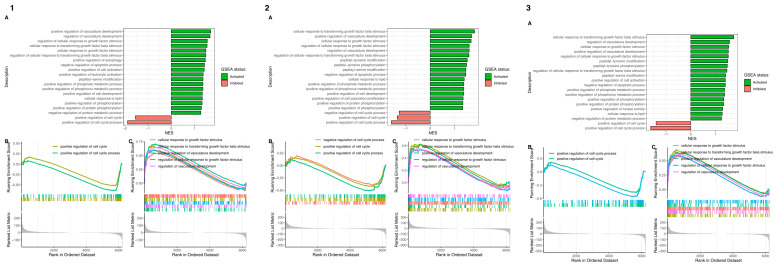
Gene Set Enrichment Analysis (GSEA) for cells at 48 (**1**), 96 (**2**), and 144 h (**3**) in comparison to the control. (**A**) The normalized enrichment score (NES) is depicted as a bar plot, indicating the enrichment of a gene set at the top of a ranked list (green indicators), while gene sets with a negative NES are overrepresented at the bottom of the gene list (red indicators). (**B**,**C**) Elaborate enrichment plots showcase the profiles of the leading ES score and the positions of genes on the rank-ordered list for the top five inhibited/activated gene sets.

Moreover, overrepresented biological processes or pathways were revealed among the differentially expressed genes. The ontological terms exhibiting the highest and lowest *p*-values underwent Gene Set Enrichment Analysis (GSEA) (Figure 6). This analysis provided insights into the functional enrichment of gene sets or predefined gene groups within the dataset. Based on the normalized expression level data, a list of significantly described terms from the Hallmark database software was generated. Across all analyzed groups, only two Gene Ontology gene groups were inhibited; “positive regulation of cell cycle process” (GO:0090068) and its parental group “positive regulation of cell cycle” (GO:0045787). Meanwhile, improved processes were observed related to the regulation of vasculature development, cellular response to transforming growth factor beta (TGF-β) stimulus, positive regulation of cell development, and cellular response to lipids.

**Figure 7:**
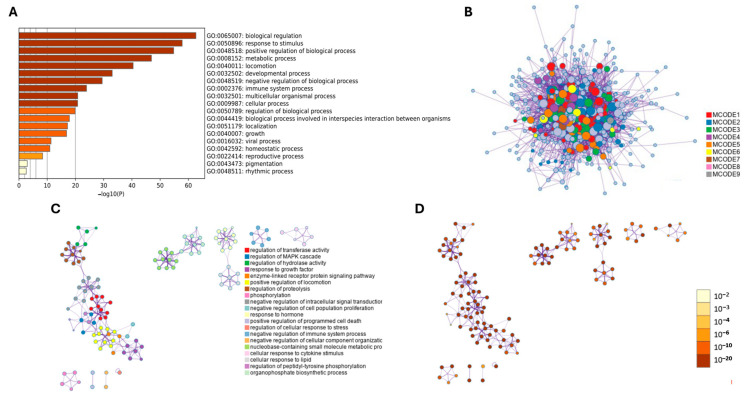
Functional enrichment analysis of differentially expressed genes (DEGs) using Metascape, categorized according to biological processes. (**A**) Bar chart displaying the top 20 clustered enrichment ontology categories, including GO and KEGG terms. (**B**) The protein–protein interaction (PPI) network was organized into the nine most notable MCODE components. In this representation, each enriched Gene Ontology (GO) term was depicted as a circular node, with its size corresponding to the number of input genes associated with that term. Additionally, the color of each node indicated its cluster identity. (**C**) Representation of the enrichment ontology clusters, with each term depicted as a circular node. The color of each node signifies the cluster identity to which the term belongs. (**D**) The enrichment network, where nodes are colored based on their *p*-values, presented in various shades as indicated in the accompanying legend. Nodes with darker colors signify higher statistical significance.

A comprehensive functional enrichment analysis, using the Metascape platform (Figure 7), identified overrepresented biological terms across all DEGs. In total, 19 top statistically enriched GO terms were identified. Among these, the top five enriched processes were biological regulation (GO:0065007, log10(P) = −62.65); response to stimulus (GO:0050896, log10(P) = −57.80); positive regulation of biological processes (GO:0048518, log10(P) = −54.78); metabolic processes (GO:0008152, log10(P) = −46.91); and locomotion (GO:0040011, log10(P) = −40.43) (Figure 7A). Subsequently, a sophisticated clustering algorithm was employed to cluster similar functional terms, to identify cohesive functional clusters within the enrichment results. These findings are presented in a network layout (Figure 7B–D), facilitating identifying related biological processes and pathways that may share common underlying mechanisms. The current analyses validated the results and reveal comparable biological processes (GO BP) with equivalent statistical significance.

**Figure 8:**
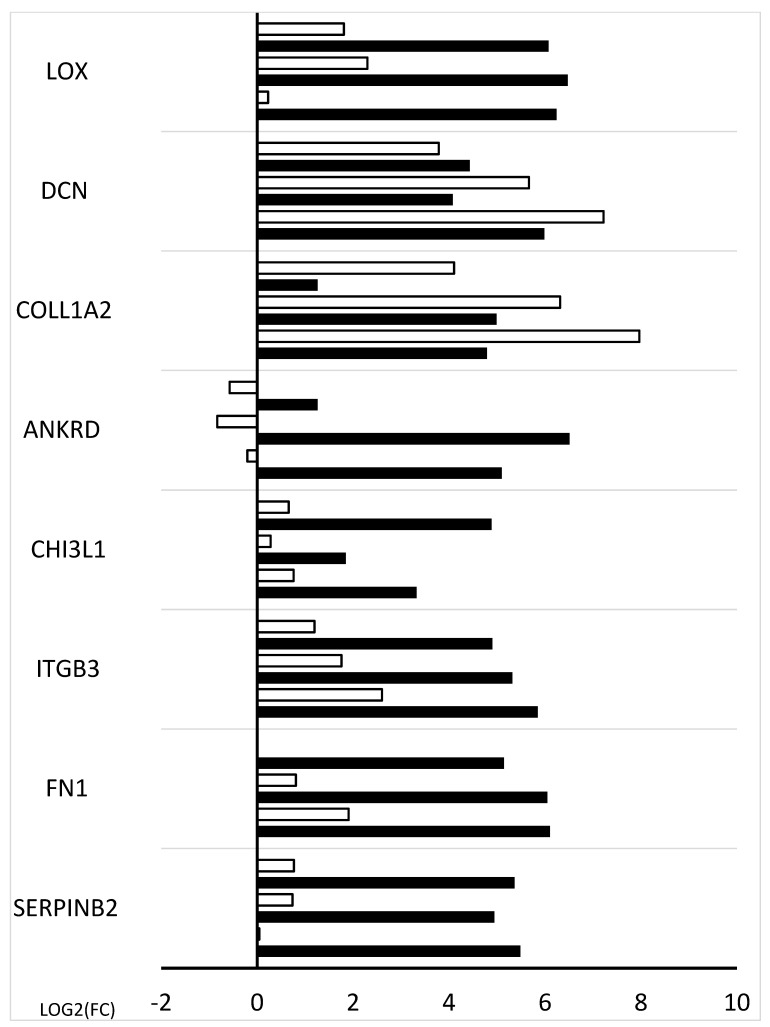
Bar graph showing validation results of microarrays of downregulated genes obtained by RT-qPCR.

The following assay quantitatively validates the microarray data using RT-qPCR. The analysis (Figure 8) shows the relative variations in transcript levels from cells before cultivation and at three distinct cell culture phases (48 h, 96 h, 144 h). All reported sample means were statistically significant (*p* < 0.05).

## 3. Discussion

The proper development and functioning of the entire reproductive system require intercellular cooperation and proper cell physiology [41,42]. GCs fulfill different functions, including modulation on signaling for energy production, formation of new blood vessels around the follicles, growth promoting and modulating their metabolism, which is driven by the fluctuating hormone levels that control oocyte maturation [43,44,45,46]. In vitro culturing reflects activity and metabolism of isolated GCs, with emphasis on cell culture as an isolated cell environment. Via extracting cells from the complex microenvironment network, in vitro could expose patterns of activity, reflecting the behavior of native tissue [47]. In vivo, GCs are influenced by hormonal signals, cell–cell communication, and interactions with other ovarian components, which tightly regulate processes such as follicular development, steroidogenesis, and ovulation [48,49]. However, in vitro culturing allows the study of the cells in a tightly controlled environment [50]. Understanding the biological mechanisms in two-dimensional culture will allow for further exploration of the holistic functioning and development of reproductive system cells.

Gene expression profile reveal that GCs undergo dynamic modulation, reflecting cellular adaptation to the environment of dynamically changing tissue. Notably, downregulation of GARNL3 and ARRDC4 was consistent across all time points (48 h, 96 h, and 144 h), suggesting these genes are likely involved in processes more active in vivo, such as cell communication and signaling with the ovarian follicular environment. GARNL3 is implicated in GTPase regulation, enabling it to act as a GTPase activator [51]. GTPases, mostly observed at a surface of intracellular membranes, are noted as a regulator of various cytosolic pathways including cell shape, intracellular signaling, vesicle forming, and intercellular transport [52]. Thus, downregulation may be an effect of reduced intracellular signaling. ARRDC4, involved in arrestin-mediated signaling, also shows diminished activity in three time points, possibly indicating decreased cellular responses to external stimuli at in vitro conditions.

In contrast, upregulation of specific genes varies depending on the time in vitro. After 48 h, a significant upregulation of LOX, FN1, ITGB3, SERPINB2, and CHI3L1 was observed. LOX is essential for collagen cross-linking, especially during ECM remodeling. As Bai et al. [53] suggests, the LOX expression in GCs positively correlates with the competence of oocytes, suggesting LOX as a potential diagnostic biomarker of oocyte quality in assisted reproduction [53]. Also, it may play a crucial role during differentiation, oocyte maturation, and also ovulation, in which the level of LOX dramatically peaks [54]. The LOX level was constantly elevated though the other time points (96 h and 144 h), without significant fluctuating over time. Furthermore, in GCs, it is demonstrated that LOX fulfills a variety of functions, influencing signaling pathways such as MAPK, ERK, and FAK, crucial for granulosa cells function [55]. Another protein with consistently elevated level is FN1, suggesting its central role in adapting to the in vitro environment, related with attempts to rebuild the structural ECM of the experiment conditions. FN1 and ITGB3 are crucial for cellular adhesion and ECM interactions, referring to the cells’ efforts to expand the culture environment [56]. FN1 is one of the genes highly expressed by maturating oocytes, thus its expression by GCs may be highly linked with intercellular granulosa cell–oocyte interactions [57]. Another gene involved in GCs’ metabolism is SERPINB2, which plays a role in protease inhibition, potentially protecting cells from excessive proteolytic damage [58]. Activation of SERPINB2 is involved in increasing GCs’ proliferation and survival rate, which are related with TNFα-Erk1/2 signaling pathway [59]. Protective role also exhibited by CHI3L1 (known as a YKL-40). Its activation is involved in inflammation and tissue repair, while upregulated expression suggests an early stress response inside the cell [60,61].

After 96 h, other genes have been upregulated. Increased level of ANKRD1, SLC1A1, and COL1A2 expression indicate further dynamics in cells’ differentiation, intercellular transport, and rearrangement of the cellular environment during in vitro culturing. As at other time points, the expression of FN1 and LOX is upregulated. ANKRD1 is taking part in an apoptosis modulation, especially linked with cisplatin sensitivity and an endoplasmic reticulum (ER) stress [62]. Its expression also promotes cells’ differentiation, as proved by Yi et al. (2022), ANKRD1 supports, i.e., osteo- and adipogenesis of human mesenchymal stem cells [63]. Elevated level of ANKRD1 may indicate differentiation potential, while elevated level of SLC1A1 suggests alterations in amino acid transport, enriching cellular metabolism [64]. Glutamine in follicular fluid coordinates ovulation. Therefore, a modified level of SLC1A1 promotes an ovulatory environment [65]. The persistent upregulation of LOX and FN1 underscores ongoing ECM remodeling, while COL1A2 reflects enhanced collagen production, further contributing to matrix restructuring [66]. Synthetizing collagen via GCs is particularly important because it supplies the structural proteins for the cytoplasmic environment of the maturing oocyte [67].

At 144 h, upregulation of LOX, FN1, DCN, ITGB3, and SERPINB2 was observed. The expression pattern indicates continued ECM regulation, enriched by DCN, which encodes decorin, a small leucine-rich proteoglycan, organizing collagen fibers and regulating growth factors signaling, indicating late-stage ECM reorganization [68]. The expression pattern of DCN, and its specific presence in theca, corpus luteum, and follicular fluid suggests a much larger role than just matrix development [69]. Also, correlation of DCN expression with progesterone level, endometriosis occurrence, and GC migration potential indicate the novel role of DCN during the preovulatory period and ovulation [70].

These changes highlight the complex and time-dependent gene expression shifts that GCs undergo, focusing on ECM remodeling, regulating the extracellular environment of ovaries, and promoting oocyte development and ovulation [71,72,73,74,75]. The intercellular GCs’ environment is controlled though exosomes, analysis of which indicate similar expression patterns. Analysis of GC-derived exosomes cargo shows that, according to the indicated genes, among the most secreted proteins are LOX, FN1, DCN, ANKRD1, SERPINB2, and ITGB2 [76].

Further analysis of protein–protein network interactions, using pathfindR, showed that the most enriched signaling pathways were proteoglycans in cancer (hsa05205), at time points 48 h and 96 h. At the point of 144 h, the emphasis of enrichment was placed on the MAPK signaling pathway. Proteoglycans, even described as cancer-related, perform a variety of functions that contribute to physiological functions during cellular and pericellular environment activity. They promote cell-to-cell and cell-to-ECM interactions, also influencing proliferation and migration [77,78]. GCs form cellular layers around the oocyte, forming the cumulus–oocyte complex, supporting oocyte growth and maturation by secreting essential factors [79]. Concurrently, theca cells migrate and proliferate to form follicular layers, contributing to structural integrity and providing follicular maturation, ovulation, and the release of a viable oocyte [80,81]. Additionally, these proteoglycans promote the secretion and metabolism of various growth factors and cytokines [82]. The mentioned processes are tightly related and essential at developing cell culture. The detected expression pattern does not have to be the result of cancerous phenotype, but the effect of proceeding cell culturing. Extended MAPK activation is observed most often during oxidative or ER stress, and it is related to improving apoptosis and autophagy in various cells, also GCs [3,83]. Activated MAPK may also indicate aging of GCs, which is supported by the expression pattern GSEA obtained in this study [84,85]. Besides that, the signaling pathway may also act as the intrafollicular mediator to stimulate the cumulus cell–oocyte interactions and oocyte maturation, proving its role in the reproductive system [86].

Another analysis of enriched genes, GSEA, indicates the enhanced activity in processes of vasculature development, regulation of genes related with TGF-β signaling pathway, positive regulation of cell development and cellular response to lipids. Expression of TGF-β-related genes, although significantly increased, was not distinctive compared to other analyzed genes (Figure 2; Figure 4). The NES protocol normalizes the raw enrichment score to account for gene set size and correlation with the expression dataset, and the number of individual genes, as captured in Figure 4. Vascular development is one of the universal processes observed during tissue development and remodeling, and also is sensitive to the TGF-β stimuli via vascular endothelial growth factor (VEGF) [87]. The expression balance of TGF-β/VEGF signaling pathway is crucial during folliculogenesis. Therefore, overexpression of angiogenic factors, such as VEGF, in early developing follicles may lead to ovarian abnormalities [88]. Physiological increase in angiogenic factors’ level is observed during folliculogenesis, antrum and corpus luteum formation, and ovulation [89]. Additionally, in the proximity of mature follicles, granulosa cells exhibit significantly increased angiogenic potential [90]. Increased exposure of GCs to angiogenic factors can improve cell proliferation and viability, as well as extracellular secretion, but may lead to the ovarian hyperstimulation syndrome, causing impaired ovulation [91,92]. Cellular response to lipids is crucial, especially for cell communication. They can be used as a signal transducer, and modulating intracellular pathways [93]. Lipids are one of the major exosomal cargos, they appear in follicular fluid, they mediate molecular pathways, and they also affect MAPK signaling [94]. The pattern of specific lipid expression is related to steroid hormones circulating, and its level changes in follicular fluid during the estrous cycle [95]. Lipids synthesis is a prominent feature during oocyte maturation. The lipids’ transport, storage, and lipolysis in the oocyte and the surrounding cumulus cells (CCs) leads to the proper development of oocyte [96]. Another evidence that the lipids level fluctuating is the decreased lipolysis activity after CCs maturation. CCs supply the developing oocyte with fatty acids [97]. Therefore, lipid metabolism in surrounding cells influence oocyte survival and maturation [98].

Potential limitations of the study may be related to the artificial in vitro model. Also, it is important not to overstate the significance of the data obtained at porcine isolated cells. Due to the polyovulation of this species, some mechanisms for selecting dominant follicles may not be activated or may exhibit species-specific characteristics. The reproductive cycles of humans and domestic pigs exhibit significant differences resulting from the distinct reproductive strategies of the two species. Humans have a menstrual cycle, characterized by regular shedding and excretion of the endometrium in the absence of fertilization. Pigs undergo an estrous cycle, in which the uterine lining is resorbed. Ovulation dynamics are also significant. Humans typically release a single cell, while pigs experience multiple ovulations, involving a dozen or more oocytes, allowing for numerous offspring in a single litter. These differences reflect distinct hormonal regulatory mechanisms, also being observed in molecular regulation.

Summarizing, metabolism and activity of GCs is variable and constantly fluctuating in vivo, but as proved, also in vitro. Interactions with surrounding cells and dynamics of the reproductive system, influence on GCs’ physiology and activity, with processes like oocyte maturation, shifting menstrual or estrous cycle phases, follicular fluid composition dynamics, and theca activity. The dynamics of cellular changes in the reproductive system are reflected in the expression pattern of specific genes in GCs. Increased expression of genes in vitro exhibits patterns observable in native tissue, and corresponds to the proper physiology of the system. During experiment, genes peaking in the phase of GCs’ maturation are observed, as well as genes regulating ECM remodeling during oocyte maturation. Also, expression patterns typical of the aging GCs population were observed. Comprising holistically time-dependent alterations of expression patterns with other studies, the noted changes align with the findings of several other researchers, similarly to Kulus et al. (2021), who observed the elevated expression of the ECM remodeling markers in GCs in vitro, which is consistent with the elevation of LOX and modulation of FN1 and ITGB3, reflecting the importance of ECM remodeling during GCs’ culturing [16]. This time-dependent remodeling potentially serves as GCs’ adaptation and communication system during in vitro culturing, also observed by Hui et al. (2017) [99]. The researchers also underlined alterations of gene expression related to energy and lipids metabolism, responsiveness to external signals, and cancer-related proteoglycans. Despite indicating a different genes expression analysis [99], the pattern of changes in cell activity and metabolism is similar. Kranc et al. (2019) observed enhanced GCs differentiation and proliferation [100]. The study develops understanding of the behavior and expression patterns of genes on the isolated GCs’ population in vitro, which may improve understanding and discovering the physiology and pathology associated with the reproductive system.

### Limitations

The study has some potential limitations that compromise clarity, reproducibility, and complexity of ovarian environment. The in vitro culture system is an inherent limitation of the study since it provides a controlled environment, but does not fully replicate the complex in vivo environment of the ovary. Furthermore, the investigation focused on gene expression at three discrete time points, potentially missing dynamic changes in gene regulation occurring at intermediate time intervals or over a longer duration. Another issue is the data validation. The results are based on transcriptomics, which measures mRNA abundance, representing the pre-translational stage, whereas proteomics quantifies protein levels, corresponding to the post-translational stage. Numerous regulatory processes occur between these levels, including differences in mRNA stability, translational efficiency, and protein degradation. Consequently, the correlation between mRNA and protein abundance is typically low (r ≈ 0.3–0.6) [101,102]. Therefore, proteomics should not be used to validate transcriptomic data but rather to provide functional insight into whether transcriptional changes translate into biological effects. The complexity of transcriptomic results analysis proves that further research is necessary, focused on the issues indicated in the limitations.

## 4. Materials and Methods

### 4.1. RNA Isolation

Total RNA was extracted from porcine GCs at the start of the experiment (0 h), as a control, and subsequently at 48, 96, and 144 h of in vitro culture using the Chomczyński-Sacchi method [103]. For microarray expression analysis, cultures were maintained in two biological replicates at each time interval.

Initially, 0.2 mL of chloroform (SIGMA-ALDRICH, St. Louis, MO, USA, Merck KGaA, Darmstadt, Germany) was added to the samples, which were mixed by inversion and agitated for 15 s, followed by a 15 min incubation at room temperature. The biphasic emulsion was subsequently separated by centrifugation at 12,000× *g* for 15 min at 4 °C. The upper aqueous phase containing RNA was carefully transferred to new Eppendorf tubes. Next, 0.5 mL of isopropanol (SIGMA-ALDRICH, St. Louis, MO, USA, Merck KGaA, Darmstadt, Germany) was added, the samples were mixed by inversion, agitated for 15 s, and incubated for 10 min at room temperature. The samples were then centrifuged at 12,000× *g* for 10 min at 4 °C. The resultant precipitate was washed with 1 mL of 75% ethanol solution (SIGMA-ALDRICH, St. Louis, MO, USA, Merck KGaA, Darmstadt, Germany), vortexed for 20 s, and centrifuged at 7500× *g* for 15 min at 4 °C. Following supernatant removal, the samples were air-dried and subsequently dissolved in 10–20 µL of DEPC-treated water (693520, Merck, Darmstadt, Germany), depending on the pellet size. RNA concentration was quantified using spectrophotometric analysis at λ = 260 nm with a NanoDrop spectrophotometer (Thermo Fisher Scientific, Waltham, MA, USA).

### 4.2. Primers Design

Primers were designed using Primer3 software (Table 1), with the exon–exon design method employed to prevent the amplification of genomic DNA fragments. The primers were also created based on the sequences of multiple transcript variants of the genes of interest from the Ensembl database [104]. For target cDNA quantification, relative quantification was performed using the 2^−ΔΔCq^ method.

### 4.3. Reverse Transcription

A quantity of 250 ng of isolated RNA, diluted in PCR-grade water to a final volume of 8 µL, was reverse transcribed using the RT2 First Strand Kit (Qiagen^®^, Hilden, Germany) in accordance with the manufacturer’s protocol. Except during incubation periods, samples were maintained on ice. To eliminate genomic DNA, 2 µL of GE (5× gDNA Elimination Buffer) was added to 1 µg of isolated RNA, followed by incubation at 42 °C for 5 min. Subsequently, the reaction mixture was prepared by combining 4 µL BC3 (5× RT Buffer 3), 1 µL P2 (Primer and External Control Mix), 2 µL RE3 (RT Enzyme Mix 3), and PCR-grade water to achieve a final volume of 10 µL. The samples then underwent two incubation steps: 15 min at 42 °C and 5 min at 95 °C. After incubation, the samples were cooled on ice, and 91 µL of H_2_O was added to each reaction.

### 4.4. Microarray Validation

The validation of the microarray data was performed using a LightCycler^®^ 96 Instrument (Roche Diagnostics GmbH, Mannheim, Germany), with cDNA synthesized during reverse transcription serving as the template. Primers were designed using Primer3Plus software (version 0.4.0; Whitehead Institute for Biomedical Research, Massachusetts Institute of Technology, Cambridge, MA, USA) based on sequences of selected transcript variants of the genes available in the Ensembl database (Table 1). The reaction mix components included the QUANTUM EvaGreen^®^ PCR Kit (5×) (Syngen Biotech, Wroclaw, Poland) as the master mix, 10 µM oligodeoxynucleotides (SIGMA-ALDRICH, St Louis, MO, USA, Merck KGaA, Darmstadt, Germany), and PCR-grade water. A total of 9 µL of the reaction mix and 1 µL of the template were added to each well of a 96-well plate. The plate was then sealed with a sealing foil, centrifuged at 1500 rpm (400× *g*) for 1 min, and placed in the thermocycler.

The microarray data were quantitatively validated using the RT-qPCR technique. For RT-qPCR validation, cultures were maintained in three biological replicates for each corresponding time interval. A bar chart was created using the RT-qPCR assay data (Figure 8). The microarray analysis results are depicted in the figure’s black bars, while the quantitative validation is shown in the white bars. Notably, the expression direction of 8 genes was measured, with only one gene, ANKRD, displaying a change in expression direction that differed from the microarray findings. This discrepancy may be due to different transcript variants present in the microarray compared to those used in the RT-qPCR primer design. The RT-qPCR result is considered more reliable because all available transcript variants of the ANKRD gene were used for primer design. Additionally, RT-qPCR is a more quantitative method compared to the qualitative method of the microarray method.

### 4.5. Microarray Data Analysis

BioConductor software (v4.1.2; R Core Team, 2021) and the associated libraries were employed for all data analyses, which were performed using the R programming language. The Robust Multiarray Average (RMA) normalization algorithm, implemented via the “Affy” library, was used to perform background correction, normalization, and calculation of expression values for the analyzed genes [105]. To visualize the total number of up- and downregulated genes, principal component analysis (PCA) was applied to the filtered dataset, with the results presented using the “factoextra” library [106]. Functional annotation and clustering of DEGs were performed using the DAVID bioinformatics tool (Database for Annotation, Visualization, and Integrated Discovery) [107]. The criteria for DEGs were defined by an absolute fold change greater than 2. Subsequently, the identified genes were mapped to corresponding GO terms, and significantly enriched GO terms were identified using the GO BP DIRECT database. *p*-values were adjusted using the Benjamini–Hochberg correction to control the false discovery rate [108]. Hierarchical clustering of DEGs was conducted, and heatmaps visualizing DEGs across comparisons were generated using the “ComplexHeatmap” library [109]. Pathway analysis was further enhanced using the PathFinder algorithm to identify and visualize interactions between DEGs and the biological pathways or processes they influence [110]. A graph-based network representation was constructed, where nodes represented biological processes and edges corresponded to the interactions between DEGs. This graph-based approach enabled the identification of gene–pathway relationships, offering insights into the underlying mechanisms of complex biological systems. One key advantage of PathFinder is its ability to uncover connections between genes and biological processes, which is particularly valuable when exploring intricate regulatory networks.

To identify functional protein partners within the input gene lists, the Metascape platform [111] was employed as a comprehensive tool for gene and protein function analysis, pathway enrichment, and protein–protein interaction (PPI) network exploration. For the PPI network analysis, the minimum interaction score was set to medium confidence (0.4). In cases where the PPI network contained more than three nodes, the Molecular Complex Detection (MCODE) algorithm was applied to identify densely connected clusters within the network that were directly associated with the input genes [112]. Each cluster detected by MCODE was assigned a distinct color based on its *p*-value, providing an additional layer of insight into the biological relevance and significance of the identified cluster.

## Figures and Tables

**Table 1:** Oligonucleotide sequences of primers used for RT-qPCR analysis.

Gene	Primer Sequence (5′-3′)		Product Size (bp)
LOX	F	GTACAACCTGAGATGCGCTG	208
	R	GCTGAATTCGTCCATGCTGT	
DCN	F	CTCTCTGGCCAACACTCCTC	155
	R	GCGGGCAGAAGTCATTAGAG	
COLL1A2	F	GTCAGACTGGTCCTGCTGGT	163
	R	GTCAGACTGGTCCTGCTGGT	
ANKRD	F	CTGCTTGAGGTGGGGAAGTA	178
	R	GTGTCTCACTGTCTGGGGAA	
CHI3L1	F	GGATGCAAGTTCCGACAGAT	202
	R	GAGGATCCCTTTCTCCTTGG	
ITGB3	F	GGATGCAAGTTCCGACAGAT	175
	R	AGTCCTTTTCCGAGCACTCA	
FN1	F	TGAGCCTGAAGAGACCTGCT	113
	R	CAGCTCCAATGCAGGTACAG	
SERPINB2	F	GGAAGAATACATTCGACTCTCCA	170
	R	TGGTCTCCGCATCTACAGAA	
ACTB	F	CCCTTGCCGCTCCGCCTTC	156
	R	GCAGCAATATCGGTCATCCAT	
GAPDH	F	CCAGAACATCATCCCTGCCT	185
	R	CCTGCTTCACCACCTTCTTG	
HPRT	F	CCATCACATCGTAGCCCTC	166
	R	ACTTTTATATCGCCCGTTGAC	

## Data Availability

The original contributions presented in this study are included in the article. Further inquiries can be directed to the corresponding author.
